# Vertical Distribution and Estimated Doses from Artificial Radionuclides in Soil Samples around the Chernobyl Nuclear Power Plant and the Semipalatinsk Nuclear Testing Site

**DOI:** 10.1371/journal.pone.0057524

**Published:** 2013-02-28

**Authors:** Yasuyuki Taira, Naomi Hayashida, Rimi Tsuchiya, Hitoshi Yamaguchi, Jumpei Takahashi, Alexander Kazlovsky, Marat Urazalin, Tolebay Rakhypbekov, Shunichi Yamashita, Noboru Takamura

**Affiliations:** 1 Department of Global Health, Medical and Welfare, Nagasaki University Graduate School of Biomedical Sciences, Nagasaki, Japan; 2 Department of Radiation Medical Science, Nagasaki University Graduate School of Biomedical Sciences, Nagasaki, Japan; 3 Department of Ecomaterials Science, Nagasaki University Graduate School of Engineering, Nagasaki, Japan; 4 Nagasaki University School of Medicine, Nagasaki, Japan; 5 Center for International Collaborative Research, Nagasaki University, Nagasaki, Japan; 6 Department of Pediatrics, Gomel State Medical University, Gomel, the Republic of Belarus; 7 Department of Microbiology, Semey State Medical Academy, Semey, the Republic of Kazakhstan; 8 Nagasaki Prefectural Institute for Environmental Research and Public Health, Omura, Japan; Dowling College, United States of America

## Abstract

For the current on-site evaluation of the environmental contamination and contributory external exposure after the accident at the Chernobyl Nuclear Power Plant (CNPP) and the nuclear tests at the Semipalatinsk Nuclear Testing Site (SNTS), the concentrations of artificial radionuclides in soil samples from each area were analyzed by gamma spectrometry. Four artificial radionuclides (^241^Am,^ 134^Cs, ^137^Cs, and ^60^Co) were detected in surface soil around CNPP, whereas seven artificial radionuclides (^241^Am, ^57^Co,^ 137^Cs, ^95^Zr, ^95^Nb, ^58^Co, and ^60^Co) were detected in surface soil around SNTS. Effective doses around CNPP were over the public dose limit of 1 mSv/y (International Commission on Radiological Protection, 1991). These levels in a contaminated area 12 km from Unit 4 were high, whereas levels in a decontaminated area 12 km from Unit 4 and another contaminated area 15 km from Unit 4 were comparatively low. On the other hand, the effective doses around SNTS were below the public dose limit. These findings suggest that the environmental contamination and effective doses on the ground definitely decrease with decontamination such as removing surface soil, although the effective doses of the sampling points around CNPP in the present study were all over the public dose limit. Thus, the remediation of soil as a countermeasure could be an extremely effective method not only for areas around CNPP and SNTS but also for areas around the Fukushima Dai-ichi Nuclear Power Plant (FNPP), and external exposure levels will be certainly reduced. Long-term follow-up of environmental monitoring around CNPP, SNTS, and FNPP, as well as evaluation of the health effects in the population residing around these areas, could contribute to radiation safety and reduce unnecessary exposure to the public.

## Introduction

On April 26, 1986, one of the most serious nuclear accidents involving radiation exposure occurred at Unit 4 of the Chernobyl Nuclear Power Plant (CNPP), located in Ukraine about 20 km south of the border with the Republic of Belarus. Significant releases of radioactive substances from Unit 4 of CNPP during the accident lasted 10 days and changes in the meteorological conditions during this period have resulted in a composite picture of contamination of vast territories [1], [2]. Radioactive contamination from CNPP spread over 40% of Europe and wide territories in Asia, North Africa, and North America [3]. Nearly 400 million people resided in territories that were contaminated with radioactivity at a level higher than 4 kBq/m^2^ (0.11 Ci/km^2^) from April to July 1986 [3]. In 2000, the total inventories of the fuel component radionuclides in the upper 30 cm of the soil layer in the 30-km Chernobyl zone in Ukraine were estimated as 0.4–0.5% of the radionuclide amounts in the CNPP Unit 4 at the moment of the accident [2].

Since August 29, 1949, more than 450 nuclear explosions, including atmospheric, above-ground, and underground tests, have been conducted at the Semipalatinsk Nuclear Testing Site (SNTS). Since the site’s closure in 1989, attention has been paid to clarifying the health effects in the population residing around SNTS [4]–[7]. According to some reports, fission products such as plutonium (Pu) and neutron-induced radioactivity were detected in the soil samples from SNTS.

The two main pathways leading to radiation exposure of the general public due to fallout are external exposure from radionuclides deposited on the ground and internal exposure through ingestion of contaminated foods produced in contaminated areas. It is extremely important to evaluate the environmental contamination and external and internal exposure risks due to nuclear disasters for radiation protection and public health.

On March 11, 2011, a 9.0-magnitude earthquake (The Great East Japan Earthquake) struck the east coast near Iwate, Miyagi, and Fukushima Prefectures, Japan. The earthquake in combination with the subsequent tsunami caused extensive damage to the Fukushima Dai-ichi Nuclear Power Plant (FNPP) and a radioactive plume derived from Units 1, 2, 3, and 4 of FNPP was dispersed in the atmosphere. The total amount of radioactive materials released into the atmosphere from FNPP corresponds to Level 7 on the International Nuclear and Radiological Event Scale (INES) by the International Atomic Energy Agency (IAEA). Although the effects of this accident are still being felt and will continue to affect the country, approximately 900 PBq of radioactive substances were emitted, a sixth of the amount of emissions from the Chernobyl accident when converted to ^131^I (half-life: 8.0 d). There are now vast stretches of land, totaling 1,800 km^2^, of Fukushima Prefecture with levels equaling a potential cumulative dose of 5 mSv/y or more (Available: http://naiic.go.jp/en/report/. Accessed 2012 Oct 25) [8]. Risks of internal exposure are extremely low because restrictions of food intake by the nation are strictly carried out after the FNPP accident (http://www.mhlw.go.jp/english/topics/2011eq/index.html. Accessed 2012 Oct 25). On the other hand, the risks of external exposure around the living space are attracting public attention due to considerable safety concerns. Although ongoing national efforts aimed at reducing the annual exposure dose closer to 1 mSv, which is the public dose limit specified by the International Commission on Radiological Protection (ICRP) in 1991, effective decontamination is not progressing smoothly around FNPP, primarily because of strong absorption of radiocesium by soil. In addition, secondary contamination of soil occurs after decontamination, creating another problem.

**Figure 1:**
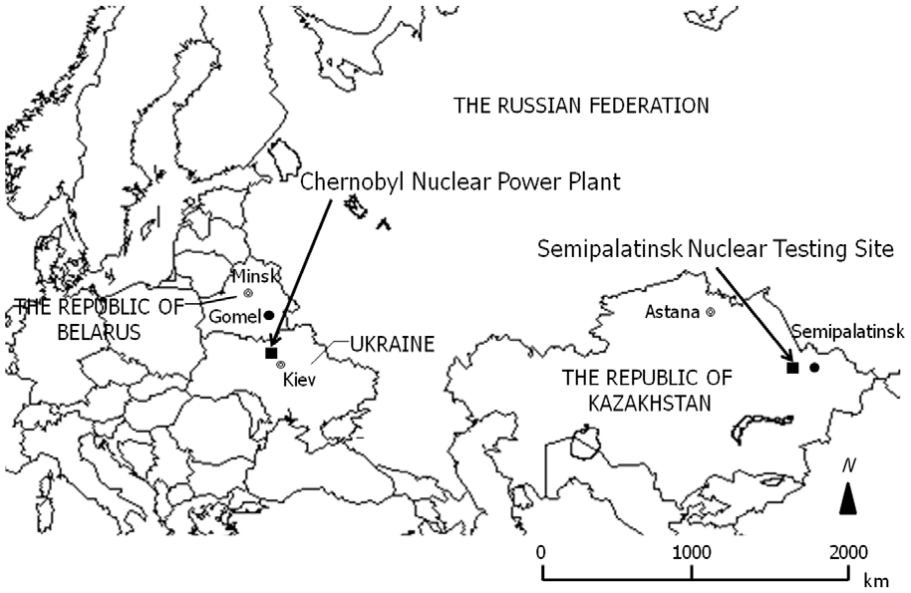
Locations around the Chernobyl Nuclear Power Plant (the Republic of Belarus, Ukraine, and the Russian Federation) and the Semipalatinsk Nuclear Testing Site (the Republic of Kazakhstan).

The evaluation of accumulated artificial radionuclides around CNPP and SNTS is extremely important for developing countermeasures such as those that will be required for future decontamination around FNPP. Therefore, to evaluate current environmental contamination and contributions from external exposure due to artificial radionuclides, concentrations of radionuclides and their vertical distribution in soil samples from areas around CNPP and SNTS were analyzed by gamma spectrometry (**Figure 1**). Furthermore, external effective doses were calculated from samples from these areas in order to estimate radiation exposure status.

## Materials and Methods

### Sample Sites

**Figure 2:**
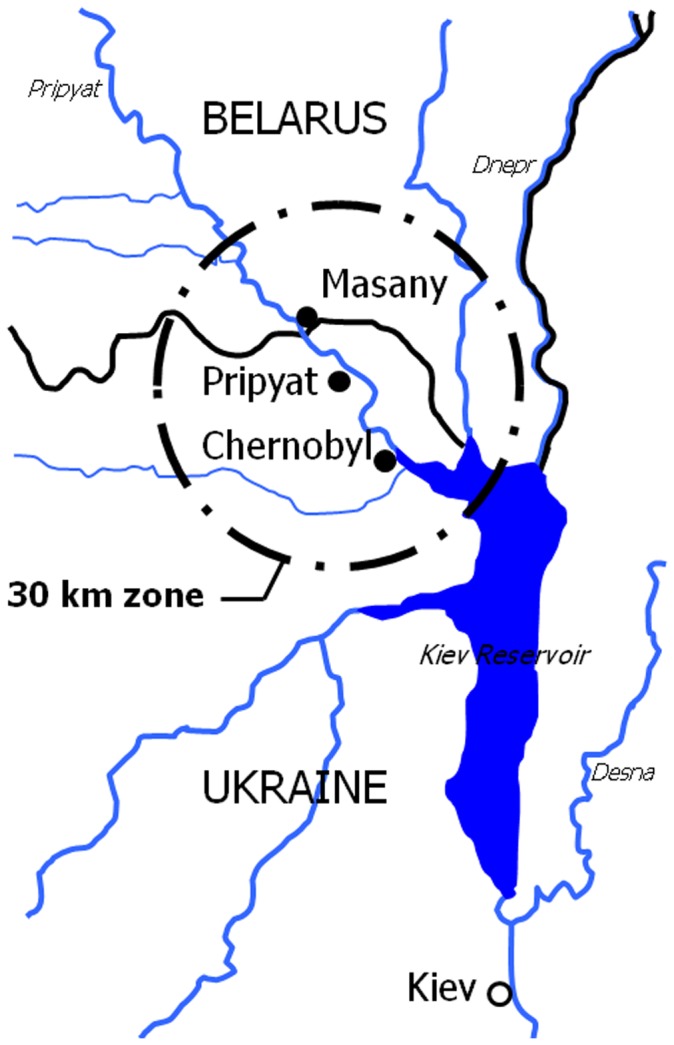
The 30-km zone around the Chernobyl Nuclear Power Plant.

Soil samples around CNPP were collected around Masany (N51° 48′, E29° 96′) in the Republic of Belarus, a fixed-point observation site approximately 8 km from the Chernobyl reactor (N51° 39′, E30° 10′), around the 30-km zone in which the ^137^Cs deposition exceeded 1,500 kBq/m^2^ (**Figure 2**) [1]. Other samples around CNPP were collected at Minsk (N53° 91′, E27° 61′) and Gomel (N52° 42′, E30° 96′) in the Republic of Belarus approximately 340 km northwest and 135 km northeast from CNPP, respectively (**Figure 1**). At the same time, air dose rates in all sample sites were monitored in air 1 m above the ground by a portable detector for the management of radiation exposure (PDR-201^®^, Hitachi-Aloka Medical, Ltd., Tokyo, Japan).

**Figure 3:**
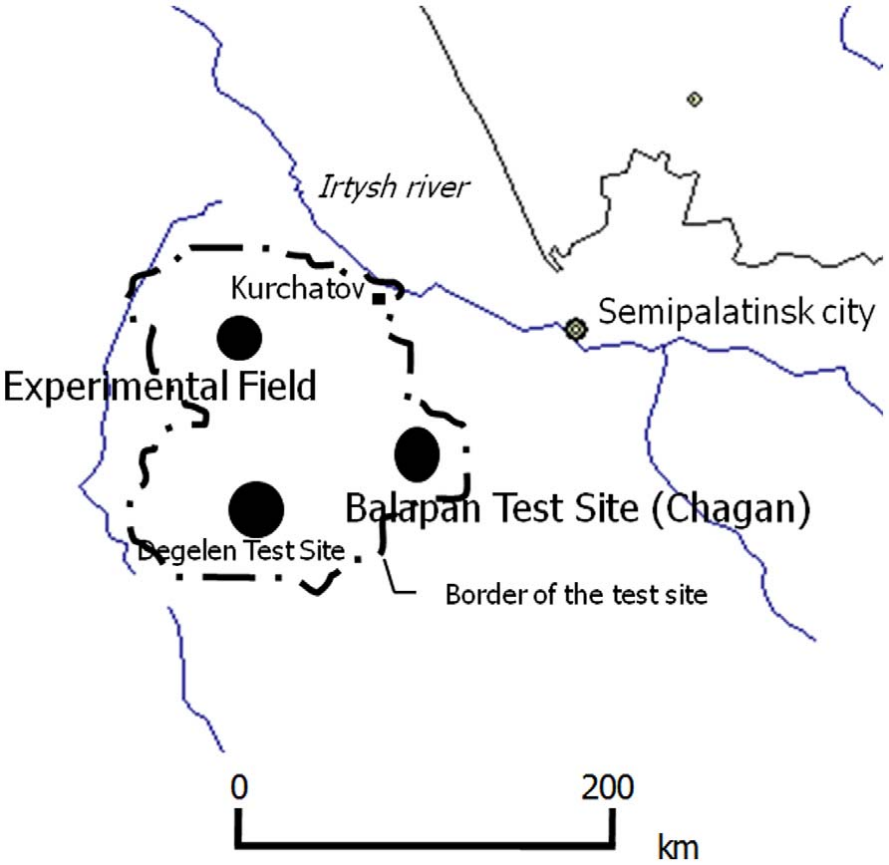
Test site around the Semipalatinsk Nuclear Testing Site.

Soil samples around SNTS were collected around the center of the explosion; the Experimental Field (N50° 20′, E77° 75′), an atmospheric and surface nuclear testing site 70 km southwest of Kurchatov, that has very high radioactivity levels and Chagan (N49° 90′, E79° 05′), known as the Balapan Test Site for underground nuclear testing in the Republic of Kazakhstan (**Figure 3**).

### Measurement of Radionuclides

For the evaluation of vertical distribution and external radiation exposure, core samples of soil (0–5 and 5–10 cm) were collected from CNPP areas between January 28 and February 3, 2012. Core samples of soil (0–5, 5–10 and 10–30 cm) were also collected from SNTS areas on August 29, 2011. Sampling of soil was carried out using soil coring at all sample sites. The size of the soil samples was 18.2 cm^2^ (a diameter of 4.8 cm) and the density of the surface soil layer ranged from 0.98 to 1.8 g/cm^3^-dry in CNPP and 1.2 to 1.6 g/cm^3^-dry in SNTS.

The mass of soil samples collected in each area ranged from 57 to 127 g. After collection, soil samples were dried in a fixed temperature dryer (105°C, 24 h) before soil samples were sieved to remove pebbles and organic materials (>2 mm).

After preparation, samples were placed in polypropylene containers and analyzed with a high purity germanium detector (CANBERRA^®^, GC2520, Canberra Industries Inc., Meriden, CT, USA) coupled to a multi-channel analyzer (Lynx, Canberra Industries Inc., Meriden, CT, USA) for 80,000 s. We set the measuring time to detect objective radionuclide levels. Gamma-ray peaks used for measurements were 59.54 keV for ^241^Am (half-life: 432.2 y), 122.06 keV for ^57^Co (271.7 d), 604.66 keV for ^134^Cs (2.1 y), 661.64 keV for ^137^Cs (30.2 y), 724.18 and 756.72 keV for ^95^Zr (64.0 d), 765.79 keV for ^95^Nb (35.0 d), 810.76 keV for ^58^Co (70.9 d), and 1173.21 and 1332.47 keV for ^60^Co (5.3 y). Decay corrections were made based on sampling data. Detector efficiency calibration for different measurement geometries was performed using mixed activity standard volume sources (Japan Radioisotope Association, Tokyo, Japan). The relative detection efficiency of this instrument was 27.8%. In the present study, we analyzed each sample at least three times, considering with “Sum effect” and “Self-Absorption”, and calculated standard errors by PASW statistics 18 software (SPSS Japan, Tokyo, Japan). Concentrations of artificial radionuclides were indicated as “counting values and ± standard errors”. Sample collection, processing, and analysis were executed in accordance with standard methods of radioactivity measurement authorized by the Ministry of Education, Culture, Sports, Science, and Technology, Japan (MEXT) [9]. All measurements were performed at the Nagasaki Prefectural Institute for Environmental Research and Public Health, Nagasaki, Japan.

### Effective Dose

After measurements, external effective doses (µSv/h and mSv/y) from soil samples were estimated from artificial radionuclide concentrations with the following formula:
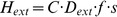
(1)in which *C* is the activity concentration of detected artificial radionuclides (^241^Am, ^134^Cs, ^137^Cs, and ^60^Co; half-life > 1y) [kBq/m^2^; estimated from radionuclide concentration in Bq/kg and collected areas of surface soil (0–5 cm)]; *D_ext_* is the dose conversion coefficient reported as the kerma-rate in air 1 m above the ground per unit activity per unit area [(µGy/h)/(kBq/m^2^)], supposing that the kerma-rate in the air and the absorbed dose rate in the air are the same value, for radiocesium with the relaxation mass per unit area (β: g/cm^2^) set to 10 due to the passage of more than 20 years after the Chernobyl accident and nuclear tests of SNTS [1.7×10^−5^ (µGy/h)/(kBq/m^2^) for ^241^Am, 2.0×10^−3^ (µGy/h)/(kBq/m^2^) for ^134^Cs, 7.6×10^−4^ (µGy/h)/(kBq/m^2^) for ^137^Cs, and 3.0×10^−3^ (µGy/h)/(kBq/m^2^) for ^60^Co, ICRU 1994] [10]; *f* is the unit conversion coefficient (0.7 Sv/Gy for effective dose rate in the body per unit absorbed dose rate in air) [11], and *s* is the decrease in the coefficient by a shielding factor against exposure with gamma rays from a deposit 1 m above the ground (0.7 under the condition of usual land) [12].

## Results

The distribution of detected artificial radionuclides in soil samples from CNPP is shown in **Table 1**. The prevalent dose-forming artificial radionuclides were ^241^Am,^ 134^Cs, ^137^Cs, and ^60^Co (these concentrations are shown in **Table 1**). Various radionuclides were especially detected near Unit 4 of CNPP. The concentrations of detected artificial radionuclides in surface soil samples around FNPP were higher than those of lower layers and the prevalent radionuclides were mainly accumulated in the surface layer.

**Table 1:** Distribution of detected artificial radionuclides in soil samples collected at the Chernobyl Nuclear Power Plant, Minsk and Gomel (Republic of Belarus).

Point	Distance^a^ (km)	Depth (cm)	Artificial radionuclides in Bq/kg-dry			
			^241^Am	^134^Cs	^137^Cs	^60^Co
CNPP (Masany)	12	Contaminated 0–5	489±3.8^b^	n.d.	63341±23	2.1±0.2,2.5±0.2
		5–10	117±1.6	n.d.	9105±8.5	n.d.
		Unknown 0–5	531±3.4	8.3±1.4	47237±20	1.6±0.3,1.0±0.2
		5–10	8.5±0.5	n.d.	753±2.4	n.d.
		Decontaminated 0–5	137±1.9	n.d.	12458±11	n.d.
		5–10	56±1.2	n.d.	4209±6.1	n.d.
	15	Contaminated 0–5	97±2.5	n.d.	18729±17	n.d.
		5–10	14±0.7	n.d.	1763±4.2	n.d.
Minsk	340		n.d.	n.d.	2.8±0.2	n.d.
Gomel	135		n.d.	n.d.	83±0.9	n.d.

a distance from Unit 4 of the Chernobyl Nuclear Power Plant.

b error shows one sigma standard deviation from counting statistics.

Samples were collected at CNPP, Minsk and Gomel, Kazakhstan during January 28 and February 3, 2012. Radionuclides were analyzed with a germanium-detector (relative detection efficiency: 27.8% by Canberra) coupled to a multi-channel analyzer for 80,000 s at Nagasaki Prefectural Institute for Environmental Research and Public Health, Nagasaki, Japan.

On the other hand, the distribution of detected artificial radionuclides in soil samples from SNTS is shown in **Table 2**. The prevalent dose-forming artificial radionuclides were ^241^Am, ^57^Co,^ 137^Cs, ^95^Zr, ^95^Nb, ^58^Co, and ^60^Co (these concentrations are shown in **Table 2**). Various radionuclides were especially detected near the center of an explosion, as with CNPP. Also, the concentrations of detected artificial radionuclides other than ^241^Am in surface soil samples around SNTS were higher than those of lower layers and those radionuclides were mainly accumulated in the surface layer.

**Table 2:** Distribution of detected artificial radionuclides in soil samples collected at the Semipalatinsk Nuclear Testing Site and Chagan (Kazakhstan).

Point	Distance^a^ (km)	Depth (cm)	Artificial radionuclides in Bq/kg-dry						
			^241^Am	^57^Co	^137^Cs	^95^Zr	^95^Nb	^58^Co	^60^Co
SNTS (Experimental Field)	Ground zero	0–5	900±6.4^b^	6079±4.8	42736±24	228±5.3, 133±5.6	15±2.9	97±2.6	347±3.5, 349±3.1
		5–10	1001±6.5	5694±4.5	39698±22	233±5.3, 124±5.6	15±2.8	83±2.3	319±3.3, 323±2.8
		10–30	590±3.7	3116±3.2	9816±11	106±3.8, 62±3.7	n.d.	48±1.9	132±2.2, 141±1.9
	1	0–5	552±2.3	25±0.3	499±2.1	n.d.	n.d.	n.d.	2.3±0.3, 2.2±0.3
		5–10	137±1.2	17±0.3	212±1.4	n.d.	n.d.	n.d.	1.9±0.4, 1.7±0.3
		10–30	318±2.0	12±0.3	138±1.3	n.d.	n.d.	n.d.	n.d.
	10	0–5	13±0.6	n.d.	26±0.6	n.d.	n.d.	n.d.	n.d.
		5–10	9.0±0.6	n.d.	24±0.6	n.d.	n.d.	n.d.	n.d.
		10–30	4.8±0.6	n.d.	12±0.5	n.d.	n.d.	n.d.	n.d.
Chagan (Balapan Test Site)		0–5	n.d.	n.d.	9.0±0.4	n.d.	n.d.	n.d.	n.d.
		5–10	n.d.	n.d.	5.9±0.3	n.d.	n.d.	n.d.	n.d.
		10–30	n.d.	n.d.	9.5±0.4	n.d.	n.d.	n.d.	n.d.

a distance from Unit 4 of the Chernobyl Nuclear Power Plant.

b error shows one sigma standard deviation from counting statistics.

Samples were collected at SNTS and Chagan, Kazakhstan on August 29, 2011. Radionuclides were analyzed with a germanium-detector (relative detection efficiency: 27.8% by Canberra) coupled to a multi-channel analyzer for 80,000 s at Nagasaki Prefectural Institute for Environmental Research and Public Health, Nagasaki, Japan.

For estimating the external effective doses, the activity concentrations in kBq/m^2^ of detected artificial radionuclides in surface soil samples (0–5 cm) around CNPP and SNTS were calculated from these radionuclides concentrations in Bq/kg (these concentrations are shown in **Table 3** and **Table 4**).

**Table 3:** Distribution of detected artificial radionuclides in soil samples collected at the Chernobyl Nuclear Power Plant, Minsk and Gomel (Republic of Belarus).

Point	Distance^a^ (km)	Depth (cm)	Artificial radionuclides in kBq/m^2^			
			^241^Am	^134^Cs	^137^Cs	^60^Co
CNPP (Masany)	12	Contaminated 0–5	28±0.2^b^	n.d.	3592±1.3	0.1±0.01, 0.1±0.01
		5–10	6.5±0.1	n.d.	509±0.5	n.d.
		Unknown 0–5	26±0.2	0.4±0.07	2322±1.0	0.1±0.01, 0.05±0.01
		5–10	0.6±0.03	n.d.	51±0.2	n.d.
		Decontaminated 0–5	5.5±0.1	n.d.	501±0.5	n.d.
		5–10	3.0±0.1	n.d.	223±0.3	n.d.
	15	Contaminated 0–5	2.3±0.1	n.d.	451±0.4	n.d.
		5–10	0.5±0.03	n.d.	68±0.2	n.d.
Minsk	340		n.d.	n.d.	0.1±0.008	n.d.
Gomel	135		n.d.	n.d.	4.6±0.05	n.d.

a distance from Unit 4 of the Chernobyl Nuclear Power Plant.

b error shows one sigma standard deviation from counting statistics.

**Table 4:** Distribution of detected artificial radionuclides in soil samples collected at the Semipalatinsk Nuclear Testing Site and Chagan (Kazakhstan).

Point	Distance^a^ (km)	Depth (cm)	Artificial radionuclides in kBq/m^2^						
			^241^Am	^57^Co	^137^Cs	^95^Zr	^95^Nb	^58^Co	^60^Co
SNTS (Experimental Field)	Ground zero	0–5	5.0±0.04^b^	34±0.03	236±0.1	1.3±0.03, 0.7±0.03	0.1±0.02	0.5±0.01	1.9±0.02, 1.9±0.02
		5–10	6.4±0.04	37±0.03	256±0.1	1.5±0.03, 0.8±0.04	0.1±0.02	0.5±0.01	2.1±0.02, 2.1±0.02
		10–30	4.0±0.03	21±0.02	67±0.1	0.7±0.03, 0.4±0.03	n.d.	0.3±0.01	0.9±0.02, 1.0±0.01
	1	0–5	6.2±0.03	0.3±0.003	5.6±0.02	n.d.	n.d.	n.d.	0.03±0.003, 0.02±0.003
		5–10	1.3±0.01	0.2±0.003	2.1±0.01	n.d.	n.d.	n.d.	0.02±0.004, 0.02±0.003
		10–30	2.2±0.01	0.1±0.002	0.9±0.01	n.d.	n.d.	n.d.	n.d.
	10	0–5	0.1±0.01	n.d.	0.2±0.01	n.d.	n.d.	n.d.	n.d.
		5–10	0.1±0.004	n.d.	0.2±0.004	n.d.	n.d.	n.d.	n.d.
		10–30	0.03±0.004	n.d.	0.1±0.004	n.d.	n.d.	n.d.	n.d.
Chagan (Balapan Test Site)		0–5	n.d.	n.d.	0.1±0.004	n.d.	n.d.	n.d.	n.d.
		5–10	n.d.	n.d.	0.1±0.003	n.d.	n.d.	n.d.	n.d.
		10–30	n.d.	n.d.	0.1±0.004	n.d.	n.d.	n.d.	n.d.

a distance from Unit 4 of the Chernobyl Nuclear Power Plant.

b error shows one sigma standard deviation from counting statistics.

The external effective doses from detected artificial radionuclides around CNPP and SNTS using Eq. (1) are summarized in **Table 5** and **Table 6**. Estimated external effective doses around CNPP were 1.3 µSv/h (12 mSv/y) in a contaminated area 12 km from Unit 4, 0.86 µSv/h (7.5 mSv/y) in a unknown area 12 km from Unit 4, 0.19 µSv/h (1.6 mSv/y) in a decontaminated area 12 km from Unit 4, and 0.17 µSv/h (1.5 mSv/y) in a contaminated area 15 km from Unit 4. Air dose rates were 0.80–4.2 µSv/h when soil samples were collected in areas around CNPP. Estimated external effective doses around CNPP were 4.2×10^−5^ µSv/h (3.7×10^−4^ mSv/y) in Minsk and 1.7×10^−3^ µSv/h (1.5×10^−2^ mSv/y) in Gomel. Air dose rates were 0.05–0.06 µSv/h when soil samples were collected in areas around CNPP.

**Table 5:** External effective doses from soil samples due to artificial radionuclides in the Chernobyl Nuclear Power Plant, Minsk and Gomel (Republic of Belarus).

Point	Distance (km)	Condition	External effective dose^a^		Air dose rate in µSv/h
			µSv/h	mSv/y	
CNPP (Manany)	12	Contaminated	1.3	12	4.2
		Unknown	0.86	7.5	3.2
		Decontaminated	0.19	1.6	0.80
	15	Contaminated	0.17	1.5	0.84
Minsk	340		4.2×10^−5^	3.7×10^−4^	0.06
Gomel	135		1.7×10^−3^	1.5×10^−2^	0.05

a External effective doses were calculated with the following formula: *H_ext_ = C•D_ext_•f•s.*

where *C* is the activity concentration of detected artificial radionuclides (^241^Am, ^134^Cs, ^137^Cs and ^60^Co; halh-life > 1y) (kBq/m^2^; calculated from radionuclide concentration in Bq/kg and collected areas of soils (0–5 cm)), *D_ext_* is the dose conversion coefficient as kerma-rate in air at 1 m above ground per unit activity per unit area ((µGy/h)/(kBq/m^2^) for detected artificial radionuclides with the value of relaxation mass per unit area 10 g/cm^2^ (ICRU 1994)), *f* is the unit conversion coefficient (0.7 Sv/Gy (UNSCEAR 2000)), *s* is the decrease in the coefficient by a shielding factor against exposure with gamma rays from a deposit at 1 m above ground (0.7 under the condition of usual land (IAEA-TECDOC-1162)).

**Table 6:** External effective doses from soil samples due to artificial radionuclides in the Semipalatinsk Nuclear Testing Site and Chagan (Kazakhstan).

Point	Distance(km)	External effective dose^a^	
		µSv/h	mSv/y
SNTS (Experimental Field)	Ground zero	9.3×10^−2^	0.79
	1	2.2×10^−3^	1.9×10^−2^
	10	8.3×10^−5^	7.3×10^−4^
Chagan (BalapanTest Site)		3.7×10^−5^	3.2×10^−4^

a External effective doses were calculated with the following formula: *H_ext_ = C•D_ext_•f•s.*

where *C* is the activity concentration of detected artificial radionuclides (^241^Am, ^137^Cs and ^60^Co; halh-life > 1y) (kBq/m^2^; calculated from radionuclide concentration in Bq/kg and collected areas of soils (0–5 cm)), *D_ext_* is the dose conversion coefficient as kerma-rate in air at 1 m above ground per unit activity per unit area ((µGy/h)/(kBq/m^2^) for detected artificial radionuclides with the value of relaxation mass per unit area 10 g/cm^2^ (ICRU 1994)), *f* is the unit conversion coefficient (0.7 Sv/Gy (UNSCEAR 2000)), *s* is the decrease in the coefficient by a shielding factor against exposure with gamma rays from a deposit at 1 m above ground (0.7 under the condition of usual land (IAEA-TECDOC-1162)).

On the other hand, estimated external effective doses around SNTS were 9.3×10^−2^ µSv/h (0.79 mSv/y) at Ground Zero (Experimental Field), 2.2×10^−3^ µSv/h (1.9×10^−2^ mSv/y) 1 km from the center of the explosion, 8.3×10^−5^ µSv/h (7.3×10^−4^ mSv/y) 10 km from the center of the explosion, and 3.7×10^−5^ µSv/h (3.2×10^−4^ mSv/y) in Chagan (Balapan Test Site).

## Discussion

Deposition in the nearby contaminated zone (<100 km) around CNPP reflected the radionuclide composition of the fuel; volatile elements, including iodine and cesium, in the form of condensation-generated particles were more widely dispersed in the far zone (from 100 km to approximately 2,000 km) [1]. The ^137^Cs deposition was highest in a 30-km radius area surrounding the reactor, known as the 30-km zone, and deposition densities exceeded 1,500 kBq/m^2^ in this zone and some areas (Gomel, Kiev, and Zhitomir regions) of the near zone to the west and northwest of the reactor [1]. According to the United Nations Scientific Committee on the Effects of Atomic Radiation (UNSCEAR), areas of ^137^Cs deposition density greater than 555 kBq/m^2^ (15 Ci/km^2^) are designated as areas of strict control following the CNPP accident on April 26, 1986 [1]. According to the 2006 IAEA report, the external doses around CNPP during 1986–2005 were about 1.2 times higher, and internal doses were about 1.1–1.5 times higher, than those obtained during 1986–1995 (depending on soil properties and applied countermeasures) [13].

In the present study, four artificial radionuclides (^241^Am, ^134^Cs, ^137^Cs, and ^60^Co) were detected in surface soil samples from contaminated and unknown areas 12 km from Unit 4 of CNPP. Additionally, these were found in high concentrations compared with the data from a decontaminated area. The value of radioactive materials released into the environment by the Chernobyl accident corresponds to Level 7 of INES by IAEA. In the present study, parts of the CNPP area may be still contaminated with artificial radionuclides derived from the nuclear disaster because current levels around CNPP were over the public dose limit of 1 mSv/y (ICRP, 1991) [14]. In particular, effective doses in a contaminated area 12 km from Unit 4, including an unknown area, were obviously high compared with an effective dose in a decontaminated area 12 km from Unit 4. In other words, these findings suggest that the environmental contamination and the effective dose on the ground are certainly decreased by decontamination such as removing surface soil, although the effective doses of sampling points around CNPP in the present study were all over the public dose limit. Thus, the remediation of soil as a countermeasure could be an extremely effective method and external exposure levels are certainly reduced. The existing remediation approaches and phytoextraction (phytoremediation) of radionuclides from contaminated soil have been examined [15], [16]. However, the remediation of soil around FNPP contaminated by artificial radionuclides is attracting considerable public attention as the public seek confirmation that the areas are safe and that external exposure risks are reduced around the living space.

Some of the detected isotopes, namely europium-152 (^152^Eu), europium-154 (^154^Eu), ^60^Co, and bismuth-217 (^217^Bi), were reported to have been produced from the stable isotopes in the ground soil around SNTS as these isotopes were activated by the neutron-induced reactions from the bomb explosions [17].

In the present study, seven artificial radionuclides (^241^Am, ^57^Co, ^137^Cs, ^95^Zr, ^95^Nb,^ 58^Co, and ^60^Co) were detected in surface soil samples near the atmospheric testing site. Moreover, these levels were high compared with data from Chagan (Balapan Test Site). However, the current levels around SNTS were below the public dose limit of 1 mSv/y. These findings suggest that the remarkable accumulation of artificial radionuclides is not confirmed in surface soil samples around SNTS, although more than 450 nuclear explosions including atmospheric, above-ground, and underground tests were conducted at SNTS from 1949 to 1989 by the former Soviet Union. Also, the results suggest that artificial radionuclides derived from atmospheric tests were widely spread and transferred after nuclear explosions.

Although the amounts of artificial radionuclides released from nuclear reactors and diffusion scales remarkably differed between CNPP and SNTS, data on the environmental radioactivity levels around CNPP and SNTS are extremely important for taking countermeasures such as decontamination against future radiation exposure in Fukushima. In the present study, short-lived radionuclides, which have a half-life of less than 1 year such as ^57^Co, ^95^Zr, ^95^Nb, and ^58^Co, were detected in soil samples. It is suggested that the soil has incorporated large amounts of radionuclides due to the nuclear disaster.

There are several limitations in the present study. Radionuclides in soil samples may be unequally distributed around FNPP because the number of sampling points was relatively small. Moreover, several radionuclides could not be analyzed by an extraction procedure, including strontium-90 (^90^Sr). However, the available depth profiles of radionuclides (fallout) in soil samples were mostly measured immediately after the nuclear disaster, although data of the initial distribution of deposited radionuclides in surface soil samples were essential for understanding their movement in the environment. In particular, radiocesium is selectively absorbed by fine soil particles, which have a greater specific surface area, and a positive relationship was found between the clay content of surface soil and the relaxation mass depth of ^137^Cs [18], [19]. Although 20 years or more have passed since the Chernobyl accident and atmospheric nuclear tests in SNTS, because the behavior of radionuclides depends on climate changes, radionuclide analysis of environmental samples by gamma spectrometry is extremely practical for the evaluation of current environmental contamination, vertical distribution, and estimated radiation dose rate. However, further investigation with detailed conditions about external and internal effective doses is needed.

In conclusion, we evaluated current environmental contamination and external radiation dose rates due to artificial radionuclides around CNPP and SNTS. Four artificial radionuclides (^241^Am, ^134^Cs, ^137^Cs, and ^60^Co) were detected in surface soil samples around CNPP and seven artificial radionuclides (^241^Am, ^57^Co, ^137^Cs, ^95^Zr, ^95^Nb,^ 58^Co, and ^60^Co) were detected in surface soil samples around SNTS. Current effective doses around CNPP were over the public dose limit of 1 mSv/y (ICRP, 1991). These levels in a contaminated area, including an unknown area, 12 km from Unit 4 were high, whereas levels in a decontaminated area 12 km from Unit 4 and a contaminated area 15 km from Unit 4 were comparatively low. On the other hand, the current effective doses around SNTS were below the public dose limit. These findings suggest that the environmental contamination and the effective dose on the ground were certainly decreased by decontamination such as removing surface soil, although the effective doses at sampling points around CNPP in the present study were all over the public dose limit. Thus, the remediation of soil as a countermeasure could be an extremely effective method not only for the areas around CNPP and SNTS but also for the areas around FNPP, and external exposure levels will be certainly reduced. Long-term follow-up of environmental monitoring around CNPP, SNTS, and FNPP, as well as evaluation of health effects in the population residing around these areas, could contribute to radiation safety and reduce unnecessary exposure to the public.
